# Comparison of EQ-5D-3L and 5L versions following operative fixation of closed ankle fractures

**DOI:** 10.1007/s11136-022-03105-2

**Published:** 2022-02-19

**Authors:** Andrew Garratt, Knut Stavem

**Affiliations:** 1 Division for Health Services, Norwegian Institute of Public Health, Oslo, Norway; 2 Institute of Clinical Medicine, University of Oslo, Oslo, Norway; 3 Department of Pulmonary Medicine, Medical Division, Akershus University Hospital, Lørenskog, Norway; 4 Health Services Research Unit, Akershus University Hospital, Lørenskog, Norway

**Keywords:** EQ-5D-3L, EQ-5D-5L, Ankle surgery, Patient-reported outcome measures, PROMs

## Abstract

**Purpose:**

To undertake the first testing and comparison of measurement properties for the EuroQol EQ-5D-3L and 5L in patients with ankle problems.

**Methods:**

The cross-sectional postal survey of 959 patients aged ≥ 18 years, who underwent surgical treatment (ORIF) for unstable and closed ankle fractures in Eastern Norway. Both the EQ-5D-3L and 5L were included in a postal questionnaire in 2015, 3–6 years post surgery. Missing data, floor and ceiling effects, and response consistency were assessed. Tests of validity included comparisons with scores for the SF-36 and widely used ankle-specific instruments. The 5L version was assessed for test–retest reliability.

**Results:**

There were 567 (59%) respondents; 501 completed both versions and 182 (61%) the 5L retest questionnaire. The 5L outperformed the 3L in tests of data quality and classification efficiency. Correlations with scores for other instruments largely met expectations, those for the 5L being slightly higher. All 5L scores had acceptable levels of reliability. For the 5L index, the smallest detectable differences for group and individual comparisons were 0.02 and 0.20, respectively.

**Conclusion:**

The 5L outperformed the 3L in terms of data quality, number of health states assessed and tests of validity. The 5L is recommended in research and other applications following surgery for ankle fracture but further testing including responsiveness to change is recommended at clinically relevant follow-up periods.

## Introduction

The EQ-5D is the most widely used short-form generic patient-reported outcome measure (PROM) suitable for economic evaluation. National scoring algorithms exist for over 20 countries, and it is available in over 170 languages [1, 2]. The EQ-5D has been widely applied in clinical and health services research, and cost per quality-adjusted life years (QALY) comparisons. National applications include the National Health Service’s Patient-Reported Outcome Measures (PROMs) programme for England [3] and Scandinavian medical registers [4–6].

Comprising just six questions or dimensions, the brevity of the EQ-5D has contributed to its application alongside ankle-specific instruments [7], which it complements through its broader focus on general aspects of health and suitability for economic evaluation. Furthermore, the availability of EQ-5D population norms, usually from a representative sample of the general population, enables greater understanding of the impact of a health problem or disease on health more generally [2]. Systematic literature searches of PubMed show that the EQ-5D has been used in 28 studies of ankle fractures and/or ankle surgery including 8 randomized controlled trials and 5 economic evaluations. For PROMs to be considered appropriate for such applications it is important that they meet widely recognized measurement criteria including reliability and validity. The scores for ankle-specific PROMs have been compared with those for the EQ-5D in testing the validity of the former [8, 9], however, there is no published evaluation of the EQ-5D measurement properties in patients with ankle fractures or ankle problems more generally.

The original version of the instrument with three levels, EQ-5D-3L or 3L, includes five important dimensions of health: mobility, self-care, usual activities, pain/discomfort and anxiety/depression. Each has three levels or response categories: no problem, some problems, severe problems. The five-level version, EQ-5D-5L or 5L, was developed by the EuroQol Group to improve the precision and responsiveness to change [3, 10]. The 5L has been widely adopted across health problems [10], including patients undergoing ankle surgery [7, 11, 12].

There is increasing evidence for the improved measurement properties of the 5L relative to the 3L, but testing in patient populations, prior to application or concurrently, is necessary [13]. Based on the findings for general and diverse patient populations, one systematic review that included 24 reports of concurrent comparisons, concluded that the 5L showed similar or better measurement properties than the 3L [10]. More recently, seven further concurrent evaluations of the two versions support these findings, in comparisons of data quality, validity and responsiveness to change [14–20]. Given its widespread application [7–9], it is important that the 5L be evaluated for measurement properties in patients with ankle fractures including concurrent evaluation alongside the 3L, to inform which version is most appropriate in these patients.

The current study is the first to report the concurrent measurement properties of the two versions of the EQ-5D administered to ankle patients as part of a retrospective cohort study 3–6 years post-surgery [7]. The study assesses measurement criteria that have been recommended and widely applied in comparisons of the two versions and follows international standards for reporting on PROMs in such a context [10, 21].

## Methods

### Data collection

This cross-sectional postal survey included 959 patients ≥ 18 years of age, who underwent surgical treatment (ORIF, open reduction internal fixation) for unstable and closed ankle fractures at two hospitals in Eastern Norway 2009–2011 [7, 22]. In January 2015, they received a postal questionnaire that included both versions of the EQ-5D and the EQ VAS with other generic and ankle-specific PROMs [7]. The accompanying letter explained the purpose of the study and that by responding to the questionnaire they gave their informed consent. The ordering of the two versions of the EQ-5D was randomized. Non-respondents received a reminder at 4.5 weeks, and at the same time a test–retest questionnaire was mailed to the first 299 respondents. The latter only included the 5L version.

The study was approved by the Norwegian Social Science Data Services (approval no. 28813/5) and the Regional Committee for Medical and Health Research Ethics, Health Region South East (approval no. 2012/384) and was conducted in accordance with the Helsinki Declaration.

### Patient-reported outcome measures

The EQ-5D instrument has a 3L or 5L descriptive system described above, as well as a visual analogue scale, the EQ VAS [3, 10], which is scored separately. Scores for both versions can be aggregated to a single score, the EQ-5D index, estimated using a scoring algorithm from a value set derived from valuation tasks undertaken with general population samples. An algorithm is not yet available for Norway and hence, current recommendations were followed for using algorithms from the UK for both versions and mapping for the 5L [23–25]. Scores for the 3L and 5L range from −0.59 to 1; 1 is the best possible health state. Negative values represent health states perceived as worse than dead. The EQ VAS assesses self-rated health on a vertical VAS, with endpoints labeled “Best imaginable health state” (100) and “Worst imaginable health state” (0) [3].

The SF-36 version 1 is a generic PROM that includes eight health domains of physical functioning, role limitations due to physical problems, bodily pain, general health, vitality, social functioning, role limitations due to emotional problems and mental health [26]. Each domain comprises two to ten items, with two- to six-point descriptive scales, that contribute to eight scores from 0 to 100 scale where 100 is the best possible health. [26, 27].

The Lower Extremity Function Scale (LEFS) has 20 items relating to physical function and daily activities with a five-point scale from ‘extreme difficulty or unable to perform’ to ‘no difficulty’ [28]. Item scores sum to a 0 to 80 score where 80 is the best possible score. The Olerud Molander Ankle Score (OMAS) has nine items relating to symptoms, physical function and daily activities [29]. Item response scales vary from two to five points, and clinical scoring reflects the level of disability. Items sum to a score from 0 to 100 where 100 is best possible. The Self-Reported Foot and Ankle Score (SEFAS) has twelve items relating to pain, limping, swelling, use of orthotics and walking [8]. The Norwegian translation followed the Swedish SEFAS [8], with a forward-backwards translation of the original English version [30] and has acceptable measurement properties [7]. The five-point scales reflect item content and sum to a 12 to 60 score where 12 is normal function. Mean imputation was used for the SF-36 and ankle-specific instruments when half or more items were completed [7, 26].

### Statistical analysis

Levels of missing data were assessed for both the 3L and 5L. To facilitate further comparisons, only respondents with complete data for the 3L and 5L were included. Response frequencies including floor (poorest level of health) and ceiling (best level of health) effects were assessed for both versions including the five items, single index, and EQ VAS. Various criteria have been proposed for acceptable levels of floor and ceiling effects including 15% [31]. More recent guidelines have not included explicit criteria, but such information is important for the interpretation of measurement properties [21, 32]. This survey was a long-term follow-up and a high proportion of respondents scoring at the ceiling was expected. Fewer were expected to score at the floor. If the additional two response categories make an important contribution, then fewer respondents might be expected to use these response categories for the 5L compared to the 3L.

Following published comparisons of the 3L and 5L, classification efficiency was assessed using Shannon’s indices of H´, which assesses the extent to which information is evenly distributed across response categories, and J´, which also takes account of the number of response categories [10].

$$H^{\prime} = \sum\limits_{i = 1}^{R} {p_{i} \ln p_{i} }$$

$$J^{\prime} = {{H^{\prime}} \mathord{\left/ {\vphantom {{H^{\prime}} {H^{\prime}\max }}} \right. \kern-\nulldelimiterspace} {H^{\prime}\max }}$$

H´ can range from 0 to 1.58 for the 3L and from 0 to 2.32 for the 5L, where higher values indicate greater efficiency. J´ can range from 0 to 1 where 1 is greater efficiency with responses evenly distributed across response categories.

Response consistency was assessed in the same manner to existing comparisons of the 3L and 5L [10, 14, 15]. There are 15 potential 3L-5L response pairs for each dimension. After transforming 3L response categories (1, 2, 3) to those for the 5L (1, 3, 5), differences of more than one category are defined as inconsistencies [10].

Reliability of index scores was assessed with the intraclass correlation coefficient within a two-way mixed effects model with absolute agreement [7, 32]. Following published recommendations [32], kappa with quadratic weighting [33] was used for assessing individual item reliability. The standard error of measurement (SEM) and smallest detectable change (SDC) were calculated. The former is the square root of the total error variance. For individuals the SDC is 1.96 × √2 × SEM and for groups, the SDC for individuals is divided by √n [32].

In tests of validity, it was hypothesized that compared to the 3L, 5L dimension scores would have higher correlations with those for the EQ VAS. Hypothesis testing was also used to assess the validity of the two sets of EQ-5D scores through comparisons with those for the SF-36 and ankle-specific instruments based on criteria for a systematic review of generic PROMs [34]:

(1) Correlations ≥ 0.60 were expected for scores assessing the same construct: EQ-5D (mobility, usual activities) and SF-36 physical functioning; EQ-5D usual activities and SF-36 role-physical; EQ-5D pain and SF-36 bodily pain; EQ-5D anxiety/depression and SF-36 mental health. This level of correlation was also expected for the EQ-5D (index, mobility, usual activities, pain/discomfort), EQ VAS and ankle-specific instrument scores. The index and EQ VAS scores were also expected to have correlations ≥ 0.60 with those for SF-36 general health and domains contributing most to physical health; physical function, role-physical, bodily pain.

(2) Correlations < 0.60 and ≥ 0.30 were expected for instrument scores assessing related but dissimilar constructs: EQ-5D (self-care, pain/discomfort) and SF-36 physical function; EQ-5D (mobility, self-care, pain discomfort) and SF-36 role-physical; EQ-5D (mobility, usual activities) and SF-36 bodily pain; all five EQ-5D dimensions and SF-36 general health; EQ-5D (mobility, usual activities) and SF-36 social function; EQ-5D anxiety/depression and SF-36 role-emotional. The index and EQ VAS scores were also expected to have correlations < 0.60 and ≥ 0.30 with those for the SF-36 contributing most to mental aspects of health: vitality, role-emotional and mental health.

(3) Correlations < 0.50 and ≥ 0.20 for scores assessing moderately related but dissimilar constructs: remaining correlations with SF-36 and ankle-specific instruments.

Stata version 15.0 (StataCorp LLC, College Station, TX) was used for statistical analyses.

## Results

### Study population

There were 567 (59.1%) respondents to the questionnaire. Table 1 shows the characteristics of the 501 respondents completing both versions of the EQ-5D. There were 182 (60.9%) respondents to the test–retest questionnaire, at a median (25th–75th percentile) of 41 (39–44) days after the first response.

**Table 1:** Characteristics of respondents completing both versions of the EQ-5D at 3–6 years follow-up (n = 501)

	N	%
Age years, mean (range)	57.2 (22.2–91.2)	
Female	284	56.7
Marital status		
Divorced/separated	69	13.9
Cohabitant/married	350	70.6
Single	41	8.3
Widowed	36	7.3
Education		
Under 10 years	142	28.7
10–12 years	188	38.1
University	164	33.2
Body mass index (kg/m^2^), median (range)	27.7 (14.4–56.7)	
Current smoker	123	24.6
Diabetes	28	5.6
Fracture classification, Weber		
A	12	2.4
B	338	67.5
C	139	27.7
Fracture, clinical features		
Uni-malleolar	263	53.1
Bimalleolar	110	22.2
Trimalleolar	122	24.6
Physical status (ASA^a^ classification)		
Completely healthy fit	178	35.5
Mild systemic disease	300	59.9
Severe systemic disease, not incapacitating	23	4.6
Postoperative length of stay in days, median (range)	7 (1–23)	
Surgery duration in minutes, median (range)	77 (7–352)	
Waiting time for surgery in days, median (range)	5 (0–64)	

^a^American Society of Anesthesiologists

### Data quality

EQ-5D dimension level missing data was similar for both versions with 527 (92.9%) and 522 (92.1%) respondents completing the five dimensions for the 3L and 5L, respectively. The EQ VAS was correctly completed by 382 (67.4%) respondents.

Table 2 shows that the vast majority of the 501 respondents completing both the 3L and 5L, reported no problems across four of the five 3L and 5L dimensions, the exception being pain/discomfort. Except for the response category denoting poorest health for the 5L self-care dimension, there were responses to all response categories across the two versions. The 501 respondents had 38, 69 and 38 states assessed by the 3L, 5L and EQ VAS, respectively.

**Table 2:** Frequencies (%) and descriptive statistics for the EQ-5D-3L, 5L and EQ VAS (n = 501)

	No problems	Slight problems	Some/ moderate problems	Severe problems	Unable/extreme
EQ-5D-3L					
Mobility	363 (72.5)		137 (13.6)		1 (0.2)
Self-care	469 (93.6)		31 (6.2)		1 (0.2)
Usual activities	360 (71.9)		134 (26.7)		7 (1.4)
Pain/discomfort	240 (47.9)		242 (48.3)		19 (3.8)
Anxiety/depression	383 (76.4)		103 (20.6)		15 (3.0)
EQ-5D-5L					
Mobility	336 (67.1)**	109 (21.8)	44 (8.8)	11 (2.2)	1 (0.2)
Self-care	465 (92.8)	26 (5.2)	6 (1.2)	4 (0.8)	0 (0.0)
Usual activities	357 (71.3)	95 (19.0)	34 (6.8)	13 (2.6)	2 (0.4)
Pain/discomfort	185 (36.9)**	230 (45.9)	62 (12.4)	20 (4.0)	4 (0.8)**
Anxiety/depression	373 (74.5)	83 (16.6)	28 (5.6)	15 (3.0)	2 (0.0)**

	Mean (SD)	Best possible	Worst possible	Number of health states^a^	Range
EQ-5D-3L indexb	0.80 (0.23)	195 (38.9)	0 (0.0)	38	− 0.25–1
EQ-5D-5L index	0.81 (0.19)**	161 (32.1)**	0 (0.0)	69	− 0.26–1
EQ VAS	80.71 (16.5)	38 (10.5)	0 (0.0)	38	20–100

^a^Number of possible health states: EQ-5D-3L = 3^5^ (243); EQ-5D-5L = 5^5^ (3125); EQ VAS = 101

^b^EQ-5D-5L index scores range from -0.57 to 1 where 1 is the best possible health state, EQ VAS scores range from 0–100 where 100 is the best possible health state

Asterisks denote statistically significant differences between the 3L and 5L using McNemar’s related-samples change test: *P < 0.05; **P < 0.01

There were very few responses to the response categories denoting the worst possible health. The greatest number of responses at this level were for the pain/discomfort and anxiety/depression dimensions for the 3L. The differences with the equivalent 5L dimensions were statistically significant. The proportion of responses to the response categories denoting the best possible health, ranged from 39–94% and from 32–93% for the 3L and 5L, respectively. Compared to the 3L, 5L responses at this level were 5–10% lower and statistically significant for mobility and pain/discomfort. For the index scores, the 5L had 7% fewer respondents scoring 1, equal to the best possible health, and this was statistically significant.

### Classification efficiency

Shannon’s H’ ranged from 0.10 (self-care) to 0.36 (pain/discomfort) and from 0.14 (self-care) to 0.50 (pain/discomfort) for the 3L and 5L dimensions, respectively. J’ ranged from 0.04 (self-care) to 0.15 (pain/discomfort) and from 0.05 (self-care) to 0.22 (pain/discomfort) for the 3L and 5L dimensions, respectively. The 5L dimensions showed mean information gain ranging from 1.23 (anxiety/depression) to 1.49 (mobility) for H’ and from 1.24 (anxiety/depression) to 1.51 (mobility) for J’.

### Response consistency

Table 3 shows response consistency across the two versions. The great majority of respondents reporting no problems for the 3L also report no problems for the 5L dimensions; 2–30% respond with slight problems for the 5L, the largest shift being for pain/discomfort. Overall, self-care and anxiety/depression had the lowest and highest levels of response inconsistency, respectively. Across the five dimensions (7–17%), most inconsistencies included respondents reporting some problems on the 3L and no problems for the 5L. Several of the other inconsistencies related to just one respondent. There were four exceptions: for mobility, 1% reported no problems for the 3L and moderate problems for the 5L; for pain/discomfort, 26.3% reported extreme problems for the 3L and moderate problems for the 5L; for anxiety/depression, 1% reported no problems for the 3L and moderate problems for the 5L; for anxiety/depression 27% reported extreme problems for the 3L and moderate problems for the 5L. The distribution of response inconsistencies was not affected by the ordering of the 3L and 5L within the questionnaire (Kruskal–Wallis test, p < 0.05).

**Table 3:** Response consistency (%) between the EQ-5D-5L and EQ-5D-3L (n = 501)

EQ-5D-3L	EQ-5D-5L				
	No problems	Slight problems	Moderate problems	Severe problems	Unable/extreme
Mobility					
No problems (n = 363)	321 (88.4)	37 (10.2)	**5 (1.4)**	**0 (0.0)**	**0 (0.0)**
Some problems (n = 137)	**15 (10.9)**	72 (52.6)	39 (28.5)	10 (7.3)	**1 (0.7)**
Unable/extreme (n = 1)	**0 (0.0)**	**0 (0.0)**	**0 (0.0)**	1 (100.0)	0 (0.0)
Self-care					
No problems (n = 469)	461 (98.3)	8 (1.7)	**0 (0.0)**	**0 (0.0)**	**0 (0.0)**
Some problems (n = 31)	**4 (12.9)**	18 (58.1)	6 (19.4)	3 (9.7)	**0 (0.0)**
Unable/extreme(n = 1)	**0 (0.0)**	**0 (0.0)**	**0 (0.0)**	1 (100.0)	0 (0.0)
Usual activities					
No problems (n = 360)	334 (92.8)	25 (6.9)	**1 (0.3)**	**0 (0.0)**	**0 (0.0)**
Some problems (n = 134)	**23 (17.2)**	69 (51.5)	33 (6.7)	9 (6.7)	**0 (0.0)**
Unable/extreme (n = 7)	**0 (0.0)**	**1 (14.3)**	**0 (0.0)**	4 (57.1)	2 (28.6)
Pain/discomfort					
No problems (n = 240)	167 (69.6)	71 (29.6)	**1 (0.4)**	**1 (0.4)**	**0 (0.0)**
Some problems (n = 242)	**18 (7.4)**	158 (65.3)	56 (23.1)	10 (4.1)	**0 (0.0)**
Unable/extreme (n = 19)	**0 (0.0)**	**1 (5.3)**	**5 (26.3)**	9 (47.4)	4 (21.1)
Anxiety/depression					
No problems (n = 383)	359 (93.7)	20 (5.2)	**4 (1.0)**	**0 (0.0)**	**0 (0.0)**
Some problems (n = 103)	**14 (13.6)**	63 (61.2)	20 (19.4)	6 (5.8)	**0 (0.0)**
Unable/extreme (n = 15)	**0 (0.0)**	**0 (0.0)**	**4 (26.7)**	9 (60.0)	2 (13.3)

Inconsistencies (marked in bold) are defined as differences of more than one between 3 and 5L response categories

### Reliability and smallest detectable change

Table 4 shows that weighted kappa for the individual 5L dimensions, and intraclass correlation coefficient for the index and EQ VAS scores, indicated good agreement between test and retest. Only the weighted kappa for the self-care dimension fell well below the criterion of 0.7 for reliability [32]. SEMs ranged from 0.19 to 0.37 for self-care and pain/discomfort dimensions, respectively. The SDC for comparisons of individuals ranged from 0.53 to 1.02, and for groups, from 0.04 to 0.08 for the same dimensions, respectively. The EQ-5D index had an SEM of 0.07, and SDCs of 0.20 and 0.02 for individuals and groups, respectively. The EQ VAS had an SEM of 7.37, and SDCs of 20.44 and 1.88 for individuals and groups, respectively.

**Table 4:** EQ-5D-5L and EQ VAS reliability, standard error of measurement and smallest detectable change (n = 164)

	Frequencies % test, retest								
	No problems	Slight problems	Moderate problems	Severe problems	Unable/extreme	Correlation/Kappa^c^	SEM^d^	SDC^e^ individual	SDC^e^ group
Mobility	72.6, 78.0	17.7, 14.0	7.9, 5.5	1.8, 2,4	–	0.83	0.29	0.80	0.06
Self-care	94.5, 90.9	4.9, 8.5	0.6, 0.6	–	–	0.57	0.19	0.53	0.04
Usual activities	81.1, 75.0	11.0, 17.7	6.1, 4.9	1.8, 2,4	–	0.75	0.34	0.94	0.07
Pain/discomfort	39.0, 47.0	47.0, 40.9	11.0, 10.4	2.4, 1.2	0.6, 0.6	0.76	0.37	1.02	0.08
Anxiety/ depression	79.3, 79.9	14.0, 15.2	4.3, 3.0	1.8, 1.2	0.6, 0.6	0.86	0.25	0.70	0.05
	Mean (SD) test		Mean (SD) retest						
EQ-5D index^a, b^	0.83 (0.17)		0.85 (0.18)			0.82	0.07	0.20	0.02
EQ VAS^b^	83.28 (14.80)		80.62 (16.19)			0.76	7.37	20.44	1.88

^a^164 (90.1%) of the 182 respondents correctly completed the EQ-5D-5L at test and retest. 118 respondents correctly completed the EQ VAS at test and retest

^b^EQ-5D-5L index scores range from -0.57 to 1 where 1 is the best possible health state, EQ VAS scores range from 0–100 where 100 is the best possible health state

^c^Intraclass correlation (index and EQ VAS), kappa with quadratic weighting (dimensions)

^d^Standard error of measurement

^e^Smallest detectable change

### Validity

Table 5 shows the correlations between the 3L and 5L dimension, index, and EQ VAS scores. Correlations between EQ-5D index scores and contributing dimensions ranged from 0.40 to 0.87, and from 0.42 to 0.88, for the 3L and 5L, respectively. Except for anxiety/depression, which had the same level for both versions, the 5L dimensions and index scores had slightly higher correlations with those for the EQ VAS. Correlations between corresponding 3L and 5L dimension scores ranged from 0.70 to 0.82 for pain/discomfort and self-care, respectively. 3L dimension scores generally had slightly higher correlations with those for the 5L than with other 3L dimension scores.

**Table 5:** Listwise Spearman correlations for EQ-5D-3L, 5L and EQ VAS scores (n = 501)

			EQ-5D-3L					
	EQ-5D-5L index	EQ VAS	EQ-5D-3L Index	Mobility	Self-care	Usual activities	Pain/discomfort	Anxiety/depression
EQ-5D-3L index	0.84	0.57						
Mobility	0.66	0.48	0.72					
Self-care	0.40	0.32	0.40	0.37				
Usual activities	0.66	0.48	0.73	0.66	0.43			
Pain/discomfort	0.68	0.43	0.87	0.54	0.20	0.53		
Anxiety/depression	0.50	0.39	0.55	0.28	0.29	0.31	0.23	
EQ-5D-5L index		0.58	0.82	0.66	0.38	0.63	0.71	0.51
Mobility	0.79	0.50	0.72	0.75	0.40	0.70	0.59	0.31
Self-care	0.42	0.35	0.40	0.39	0.82	0.43	0.22	0.31
Usual activities	0.74	0.49	0.71	0.71	0.44	0.78	0.57	0.33
Pain/discomfort	0.88	0.48	0.72	0.56	0.31	0.57	0.70	0.30
Anxiety/depression	0.56	0.39	0.45	0.27	0.27	0.29	0.19	0.81

Undertaken separately for the EQ-VAS, which had a lower number of respondents (n = 363)

All correlations are significant (p < 0.01)

Table 6 shows that correlations of ≥ 0.6 were found between 3L, 5L and SF-36 scores assessing very similar aspects of health. Correlations with the three ankle-specific scores were of a similar magnitude for those of the EQ-5D assessing overlapping aspects of health: mobility, usual activities, and pain/discomfort. Correlations of ≥ 0.6 were also found for the two index scores and those for SF-36 general health, SF-36 domains mostly related to physical health and the ankle-specific scores. Two of the correlations for both index scores were slightly lower than expected (0.58–0.59). For the scores assessing related but dissimilar constructs, all but two correlations were in the range < 0.60 and ≥ 0.30. The exception was pain/discomfort at 0.61 for both EQ-5D versions. All but one of the remaining correlations (3L pain/discomfort and SF-36 mental health) were in the expected range of < 0.50 and ≥ 0.20 for scores assessing moderately related but dissimilar constructs. There were 11 correlations of the same size but the majority (44/66) were slightly higher for the 5L compared to the 3L scores. The differences in correlation were largest for the ankle-specific instruments. Compared to both index scores, EQ VAS scores had lower correlations with those for the SF-36 and ankle-specific instruments. These were slightly lower than expected for the ankle-specific instruments and SF-36 role-physical and bodily pain domains.

**Table 6:** Listwise Spearman correlations with SF-36 and ankle instrument scores (n = 451)

	SF-36 domains										
	Physical function	Role-physical	Bodily pain	General health	Social function	Vitality	Role-emotional	Mental health	LEFS	OMAS	SEFAS
EQ-5D-3L index	0.73	0.59	0.74	0.59	0.51	0.52	0.39	0.37	0.73	0.80	0.79
Mobility	0.64	0.56	0.60	0.49	0.41	0.42	0.30	0.26	0.65	0.67	0.65
Self-care	0.36	0.35	0.31	0.31	0.26	0.38	0.29	0.25	0.35	0.34	0.33
Usual activities	0.63	0.64	0.64	0.51	0.42	0.49	0.35	0.30	0.62	0.62	0.63
Pain/discomfort	0.61	0.42	0.67	0.44	0.33	0.30	0.20	0.16	0.64	0.73	0.75
Anxiety/depression	0.35	0.35	0.32	0.40	0.45	0.53	0.56	0.58	0.29	0.30	0.28
EQ-5D-5L index	0.72	0.58	0.75	0.59	0.52	0.55	0.42	0.40	0.74	0.80	0.80
Mobility	0.68	0.59	0.66	0.54	0.44	0.46	0.36	0.27	0.69	0.74	0.74
Self-care	0.39	0.36	0.31	0.32	0.26	0.37	0.28	0.25	0.38	0.34	0.35
Usual activities	0.67	0.62	0.64	0.49	0.40	0.47	0.37	0.26	0.67	0.67	0.70
Pain/discomfort	0.61	0.47	0.71	0.49	0.41	0.40	0.29	0.28	0.66	0.75	0.78
Anxiety/depression	0.35	0.35	0.33	0.40	0.47	0.58	0.58	0.59	0.30	0.28	0.25
EQ VAS^a^	0.61	0.50	0.55	0.60	0.47	0.62	0.33	0.47	0.54	0.52	0.50

All correlations are significant (p < 0.01). Underlined coefficients are those expected to have the highest level of correlation ≥ 0.6

*LEFS* Lower Extremity Function Scale, *OMAS* Olerud Molander Ankle Score, *SEFAS* Self-Reported Foot and Ankle Score

^a^Undertaken separately for the EQ-VAS, which had a lower number of respondents (n = 331)

## Discussion

The study found that the two EQ-5D versions had satisfactory data quality, reliability, and validity. In general, the differences in performance of the two versions was not large, but the 5L performed slightly better across several important measurement criteria. The concurrent nature of the evaluation reported here represents the strongest available evidence for choosing the 5L version in long-term follow-up after ankle surgery.

Across 5L dimensions, respondents used four or five response categories and hence described a greater range of health states than for the 3L (69 compared to 38). Very few respondents had dimension scores corresponding to the lowest possible health, but the 5L had fewer such responses for pain/discomfort and anxiety/depression, the former being an important dimension for ankle fracture. One systematic review of studies comparing the two versions across diverse illness groups and the general population, reported similar improvements for the 5L, the largest being for the pain/discomfort dimension [10].

Given the long-term follow-up nature of the survey, a high proportion of respondents scoring at the best possible levels of health, was expected. There was little difference between the two versions for three dimensions, but responses at the ceiling were up to 11% lower for the 5L compared to the 3L for the dimensions of mobility and pain/discomfort, and statistically significant. These are important aspects of health in this group of respondents, including long-term follow up. The systematic review of 3L and 5L comparisons did not include long-term follow-up populations, and larger differences in ceiling effects were found; up to 17% of rmobility and 30% for self-care dimensions [10]. Statistically significant reductions in ceiling effects for the 5L compared to the 3L have been reported by more recent studies, with pain/discomfort often being the largest [19, 35–38].

Shannon’s indices showed that the 5L outperformed the 3L in tests of classification efficiency. These results follow the findings of a systematic review based on 14 studies [10] and more recent studies reporting tests of classification efficiency [14, 15, 19, 20, 35–37, 39, 40].

The very few responses to the lowest levels of health limited the assessment of response consistency across the 3L and 5L. The greatest proportion of inconsistencies related to pain/discomfort and anxiety/depression where up to 5 respondents reported extreme problems on the 3L and moderate problems on the 5L. This was followed by the most obvious pattern of inconsistencies across all five dimensions for respondents reporting some problems on the 3L and no problems on the 5L. There was no evidence that the ordering of the 3L and 5L within the questionnaire affected the level of response inconsistencies.

The test–retest design was limited to the 5L, which was considered appropriate given that evidence from a range of general and patient populations supports its application in preference to the 3L [10, 14–20]. There was no evidence for systematic differences between test and retest scores. The levels of kappa for dimension scores, and correlation for index and EQ VAS scores, largely met the 0.7 criterion [32] and were higher than most estimates reported by a systematic review of EQ-5D measurement properties [10]. The dimension of self-care was below the criterion, but it had the lowest SEM across dimensions. Reliability levels often limit the interpretation of single items, here in the form of EQ-5D dimension scores, at the group and particularly the individual level. These results were no exception but are satisfactory.

Along with the EQ-5D, the SF-36 is the most widely tested and used PROM [13, 27]. Being a health profile measure, which assesses several important aspects of health, further enhances its use in tests of validity that included EQ-5D dimension as well as in index scores. The inclusion of three widely used ankle-specific instruments [7] further contributed to validity testing. With very few exceptions, the expected correlations were found. Overall, the statistically significant correlations show that the EQ-5D is picking up adverse and other aspects of health across instruments that are widely used in ankle research and that the 5L improves upon the 3L in this respect.

## Study strengths and limitations

The concurrent nature of the 3L and 5L comparison is an important study strength, which gives the best available evidence for comparative measurement performance [13]. Moreover, the ordering of the questionnaires was randomized so that half the respondents completed the 3L first, and half, the 5L first. The inclusion of widely used generic and ankle-specific PROMs was a further strength, which allowed extensive testing for validity. The study followed widely recognized recommendations for assessing the measurement properties of PROMs in general [21, 32] and the EQ-5D [10].

The main study limitation stems from it being a long-term follow-up. It is important that the EQ-5D is assessed for measurement properties at other clinically important follow-up periods. Given the results of this and other studies [10, 14–20, 35–40], further testing should focus on the 5L. This limitation also meant that it was not possible to assess the EQ-5D along with the other instruments, for responsiveness to change. This is an important measurement property that further informs the selection of instruments for evaluative studies including clinical trials [7, 21]. The 59% response rate to the survey questionnaire is acceptable, but some statistically significant differences between respondents and non-respondents were found [22].

Limitations of the test–retest design include the six-week interval between test and retest necessitated by practical considerations, and absence of health transition questions. The EQ-5D asks about health today, and hence, the reliability estimates produced by this study might well be biased due the study design. Intervals of between one and three weeks were found across previous studies assessing the reliability of the EQ-5D [10]. Transition questions, which focus on relevant aspects of health, are widely used in test–retest studies as a means of identifying respondents whose health has changed between test and retest [41]. The absence of such a question is of particular importance given the six-week interval and changes in health that may have taken place for some respondents. Given that the EQ-5D focuses on health today, the removal of such respondents from the analysis may have contributed to improved test–retest estimates.

## Conclusions

The EQ-5D is the most widely used short generic instrument suitable for use in economic evaluation including cost per QALY calculations. It is widely used in patients with ankle fractures including clinical trials of surgery, but there is limited evidence supporting its application. This is the first study to test the measurement properties of either the 3L or 5L version of the instrument, following surgery for ankle fracture and in ankle problems more generally. The 5L version is increasingly used and hence, this concurrent evaluation of both versions is timely. Findings following testing for data quality, reliability and validity support the use of the 5L in preference to the 3L version but further testing, including responsiveness to change, is recommended at clinically relevant follow-up periods.

## Data Availability

The datasets used and/or analyzed during the current study are available from the corresponding author on reasonable request.

## Abbreviations

BMI: Body Mass Index
ICC: Intraclass correlation coefficient
LEFS: Lower extremity function scale
OMAS: Olerud molander ankle score
ORIF: Open reduction internal fixation
PROMs: Patient-reported outcome measures
SDC: Smallest detectable change
SEFAS: Self-reported foot and ankle score
SEM: Standard error of measurement
EQ-5D: EuroQol EQ-5D
